# Sex-Biased miRNAs in the Gonads of Adult Chinese Alligator (*Alligator sinensis*) and Their Potential Roles in Sex Maintenance

**DOI:** 10.3389/fgene.2022.843884

**Published:** 2022-03-31

**Authors:** Meng-Yuan Hu, Jun Yu, Jian-Qing Lin, Sheng-Guo Fang

**Affiliations:** ^1^ MOE Key Laboratory of Biosystems Homeostasis and Protection, State Conservation Centre for Gene Resources of Endangered Wildlife, College of Life Sciences, Zhejiang University, Hangzhou, China; ^2^ Guangdong Provincial Key Laboratory of Marine Biotechnology, Institute of Marine Science, Shantou University, Shantou, China

**Keywords:** Chinese alligator, miRNA, gonads, sex maintenance, sexual dimorphism

## Abstract

MicroRNA (miRNA) is a category of single-stranded non-coding small RNA (sRNA) that regulates gene expression by targeting mRNA. It plays a key role in the temperature-dependent sex determination of Chinese alligator (*Alligator sinensis*), a reptile whose sex is determined solely by the temperature during the incubation period and remains stable thereafter. However, the potential function of miRNAs in the gonads of adult Chinese alligators is still unclear. Here, we prepared and sequenced sRNA libraries of adult female and male alligator gonads, from breeding (in summer) and hibernating (in winter) animals. We obtained 130 conserved miRNAs and 683 novel miRNAs, which were assessed for sex bias in summer and winter; a total of 65 miRNAs that maintained sex bias in both seasons were identified. A regulatory network of sex-biased miRNAs and genes was constructed. Sex-biased miRNAs targeted multiple genes in the meiosis pathway of adult Chinese alligator oocytes and the antagonistic gonadal function maintenance pathway, such as *MOS*, *MYT1*, *DMRT1*, and *GDF9*. Our study emphasizes the function of miRNA in the epigenetic mechanisms of sex maintenance in crocodilians.

## Introduction

Sexual dimorphism is a phenomenon, in animals that exhibit sexual reproduction, in which there are differences between females and males (Lande, 1980). Although females and males have almost identical autosomal genomes, they have clear differences in morphology, physiology and behavior. Therefore, formation of sexual dimorphism is associated with the sex-biased gene expression triggered either by sex-determination genes or by environmental factors such as population density or temperature. As a type of small RNA (sRNA) that targets mRNA to regulate gene expression, microRNA (miRNA) is involved in the formation of sexual dimorphism (Morgan and Bale, 2012). Due to different developmental pathways, male and female gonads show clear morphological and functional sexual dimorphism. They are often used as target organs for studying miRNA expression in mammals (Torley et al., 2011; Real et al., 2013; Guo L. et al., 2017), fish (Presslauer et al., 2017; Fu et al., 2020; Li et al., 2020), and birds (Bannister et al., 2009; Luo et al., 2012; Jiang et al., 2018). However, in reptiles, specific temperature-dependent sex determination (TSD) is instead the focus. For example, a study on the Reeves’ pond turtle (*Mauremys reevesii*) has suggested that miRNAs have crucial function in sex maintenance after gonad maturation (Xiong et al., 2020). In the gonads of the Chinese soft-shelled turtle (*Pelodiscus sinensis*), a regulatory network was constructed, in which the differentially expressed miRNAs (DEmiRs) and long non-coding RNAs target many genes related to gonadal development (Ma et al., 2020).

Sex reversal is a physiological state in which phenotypic- and genotypic sex are inconsistent (Baroiller and D’Cotta, 2016). In animals exhibiting sex reversal, the initial sex is determined by its genetic factors, and, in subsequent life history, sex reversal occurs due to factors such as environment, hormones, and physiological state. Sex reversal occurs more frequently in species that have both genotypic- and environmental sex determination, including the half-smooth tongue sole (*Cynoglossus semilaevis*) (Shao et al., 2014), montane scincid lizards (*Bassiana duperreyi*) (Radder et al., 2008), and Nile tilapia (*Oreochromis niloticus*) (Sun et al., 2016). However, for crocodilians, whose sex determination mechanism is TSD-only, sex remains irreversibly stable after the temperature sensitive period (TSP) (González et al., 2019). There are indications that the fate of the gonads is not purely established by antagonistic signaling-pathway competition between male and female in the embryonic period, but also needs to be actively maintained in adulthood to prevent sexual reprogramming (Uhlenhaut et al., 2009; Matson et al., 2011; Liu et al., 2017).

As an endangered alligator unique to China, the Chinese alligator (*Alligator sinensis*) is known as a “living fossil” (Pan et al., 2019). Like the other 22 extant crocodilian species in the world, Chinese alligators have no sex chromosomes; temperature during the TSP determines their sex, and it remains stable throughout the subsequent life history (Pieau et al., 1999). Therefore, the Chinese alligator is a suitable biological model to study temperature-dependent sex determination, differentiation, and maintenance. Our previous studies on the gonads of the Chinese alligator have mainly focused on the process of sex differentiation in the embryonic stage (Lin et al., 2018), the importance of hibernation to ovarian development (Lin et al., 2020), and the genetic and epigenetic mechanisms of sex maintenance (Lin et al., 2021). The default sex of the Chinese alligator embryo is female and the male determination is triggered by high temperature through calcium signals in the middle of the TSP, while miRNA also participates in the regulation of this process (Lin et al., 2018). The *DMRT1* and steroid biosynthesis pathways in adult Chinese alligators show male-biased expression and maintain male sex (Lin et al., 2021). However, the roles of miRNA in sexual dimorphism and gonadal sex maintenance in adult Chinese alligators is still unclear.

Here, we constructed a miRNA expression profile of the Chinese alligator testes and ovaries, aiming to establish an mRNA-miRNA regulatory network and identify key miRNAs involved in sex determination and maintenance. These data will improve the epigenetic mechanism of sex determination, differentiation, and maintenance of crocodilians from embryo to adulthood.

## Materials and Methods

### Sample Collection

All procedures with the alligators were approved by the State Forestry Administration of the People’s Republic of China [Forest Conservation Permission Document (2014–1545)] and the Animal Ethics Committee of Zhejiang University (ZJU 2015-154-13). All four alligators were obtained from the Changxing Yinjiabian Chinese Alligator Nature Reserve in 2015. The samples were collected from hibernating and breeding-season adult Chinese alligators in winter and summer, respectively. Testis (W_TES) and ovary (W_OVA) in winter and testis (S_TES) and ovary (S_OVA) in summer were collected after ketamine anesthetic (5–10 mg/kg) was injected into their tails. Bloodletting and dissection were performed when they were under deep anesthesia. The gonads were extracted and put into liquid nitrogen for quick freezing, and then transferred to −80°C until use.

### RNA Extraction, sRNA Library Preparation and Sequencing

Total RNA was extracted from gonads using Trizol RNA Isolation Kit (Invitrogen, Waltham, MA) for the construction of the sRNA libraries W_TES, W_OVA, S_TES, and S_OVA. RNA integrity was assessed using the RNA Nano 6000 Assay Kit of the Agilent Bioanalyzer 2,100 system (Agilent Technologies, Santa Clara, CA), and the library was constructed for the qualified total RNA (RIN ≥8.0). For each sample, 3 µg of RNA was used to construct sRNA libraries using an NEBNext^®^ Multiplex Small RNA Library Prep Set for Illumina^®^ (New England Biolabs, Ipswich, MA), according to the manufacturer’s instructions. Then, the library preparations were sequenced on an Illumina Hiseq 2,500/2000 platform and 50 base pair single-end reads were generated.

### Sequencing Data Analysis

Clean reads were obtained by removing reads with adapter contaminants, containing poly-N, and low-quality reads from raw reads. Clean reads of 18–35 nucleotide (nt) in length were mapped to the Chinese alligator reference genome (GenBank accession number GCA_000455745.1) without mismatch using Bowtie version 2.2.3 (Langmead and Salzberg, 2012). Mirdeep2 (Friedländer et al., 2012) and miREvo (Wen et al., 2012) software were integrated to identify the miRNA of Chinese alligator and submit them to the Rfam database (Kalvari et al., 2018) for comparison to identify conserved miRNA (miRNA existing in at least one other species) for miRNA family analysis. TPM (number of transcripts mapped to miRNA per million transcripts) was used to standardize the expression of miRNA. The R software package DEGseq version 1.12.0 (Wang et al., 2010) was used to calculate the differential expression of miRNA between the two sets of samples. Expression data were calculated relative to the testicular data. DEmiR was defined as miRNA that demonstrated an absolute log_2_-fold change >1 and adjusted *p* (*q*) < 0.01. Sequences with male-bias therefore displayed a negative log_2_-fold change.

Three software platforms, namely miRanda, PIPT, and RNAhybrid (Rehmsmeier et al., 2004; Kertesz et al., 2007; Betel et al., 2010), were used to predict potential target genes of DEmiRs. Subsequently, Gene Ontology (GO) annotation and Kyoto Encyclopedia of Genes and Genomes (KEGG) pathway analysis were carried out. GOseq (Young et al., 2010) and KOBAS software (Mao et al., 2005) were used for GO annotation and KEGG pathway analysis, respectively. A *p*-value ≤ 0.05 was used to identify significantly enriched GO terms and KEGG pathways.

## Results

### sRNAs Sequencing and Assembly

**FIGURE 1:**
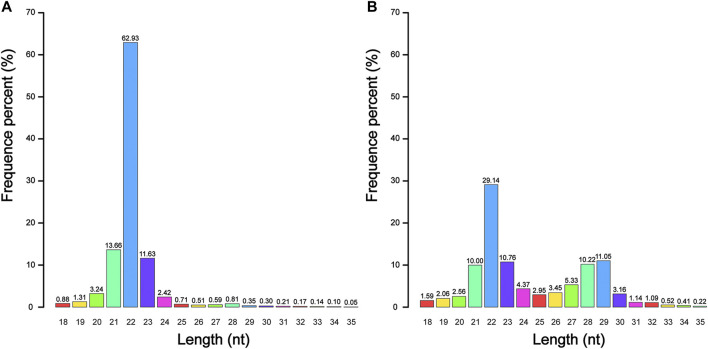
The nucleotide (nt) length distribution of small RNA in adult Chinese alligator ovaries **(A)** and testes **(B)** sampled in summer.

A total of 51 704 167 raw reads were generated from four sRNA-seq libraries, and 50 697 427 (98.05%) of them were classified as clean reads. Among the clean reads, 47 171 540 (93.05%) reads were 18–35 nt in length, of which 43 213 175 (85.24%) reads could be mapped to the reference genome for subsequent analysis (Supplementary Table S1). We found that the number of unique reads in testes was much higher than that in ovaries, which indicated that the types of reads in testes were more abundant and specific. We also found that whether in summer or winter, sRNA of 22 nt accounted for a high proportion in all four samples, with an average abundance of 43.39%, indicating that miRNA was stably expressed in the gonads of the Chinese alligator. However, the proportion of sRNA of 26–32 nt in testes was higher than that in ovaries, suggesting that Piwi-interacting RNA (piRNA) may play a more important role in the functional maintenance and development of testes than in ovaries (Figure 1). The size distribution in winter was essentially the same as that in summer (Supplementary Figure S1).

After removing other non-coding RNA and repeat sequences (Supplementary Table S2), 813 mature miRNAs were identified in the Rfam database (Supplementary Table S3). Among them, 130 conserved miRNAs were obtained, and the other 683 miRNAs were specific to the Chinese alligator. Active expression was observed for 97.69% (127/130) of conserved miRNAs, while 67.94% (464/683) of Chinese alligator specific miRNAs were actively expressed, and the total number of actively expressed miRNAs was 591 (Table 1).

**TABLE 1:** MicroRNAs identified using sRNA sequencing of Chinese alligator gonads.

	All	Actively expressed
Conserved	130	127
Specific to Chinese alligator	683	464
Total	813	591

### Sex Bias Analysis of miRNAs

**FIGURE 2:**
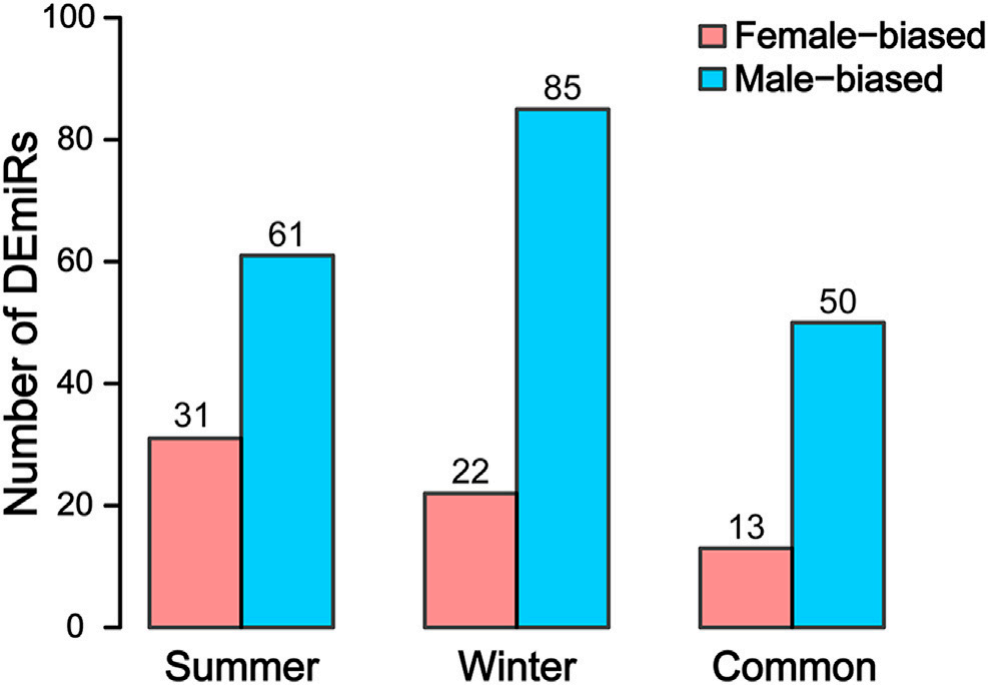
Female- and male-biased differentially expressed miRNAs (DEmiRs) in adult Chinese alligator gonads sampled in summer and winter.

In summer, there were 61 male-biased and 31 female-biased miRNAs. In winter, there were 85 male-biased and 22 female-biased miRNAs. Among them, 65 miRNAs maintained their sex bias in both seasons, of which 63 maintained stable male-biased (50) or female-biased (13) expression in both seasons (Figure 2). Two miRNAs (miR-133a and miR-144) showed inverted sex-biased patterns between the two seasons.

### Family Analysis of Sex-Biased miRNAs

**FIGURE 3:**
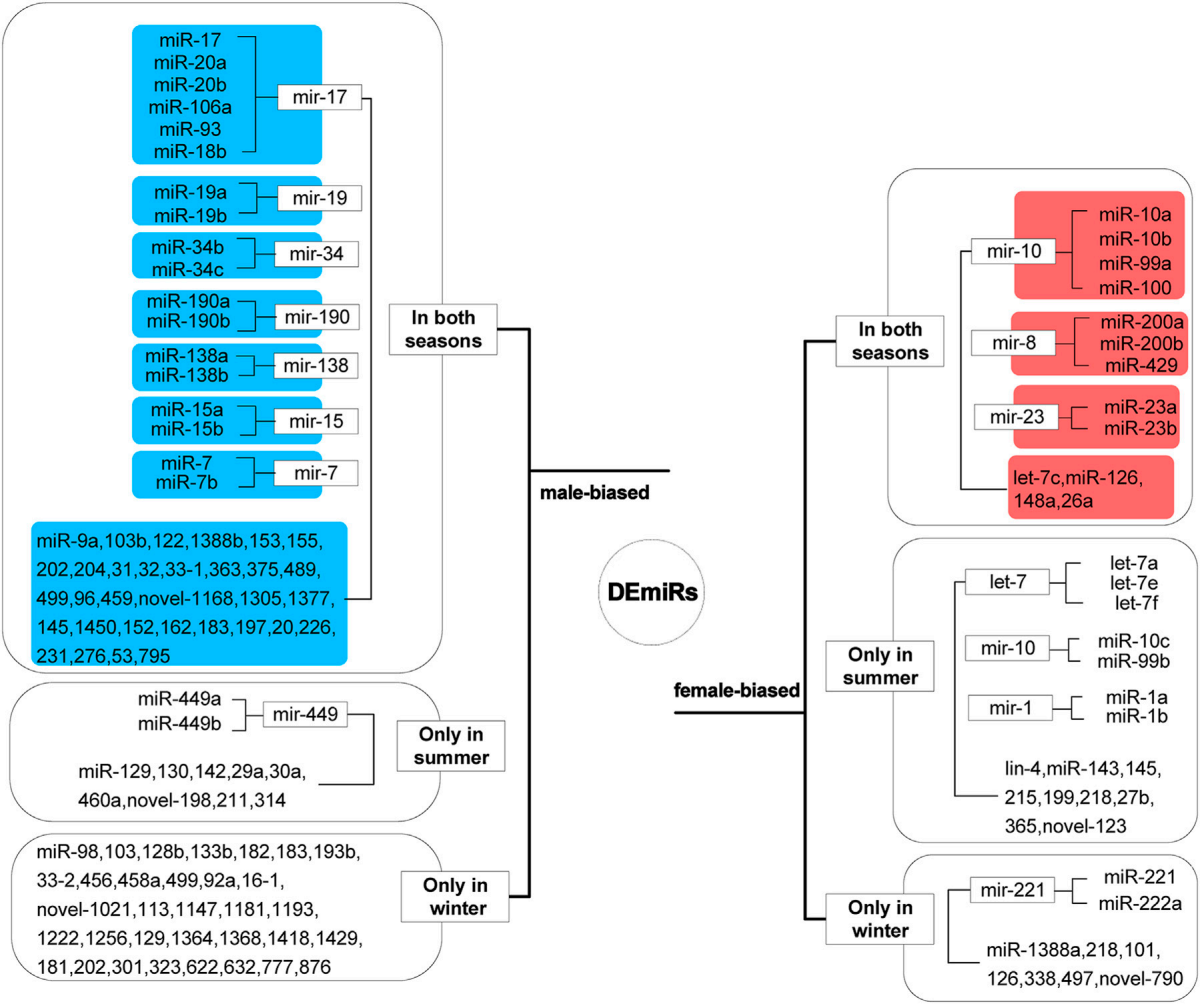
Sex-biased differentially expressed miRNAs (DEmiRs). Sequences that were similarly biased in summer and winter are highlighted in blue and red, for male- and female-bias respectively.

The 134 DEmiRs, of which 95 were conserved, belonged to 61 miRNA families. Two miRNAs (miR-133a and miR-144) with inverted expression in summer and winter were excluded, and the remaining 132 miRNAs were categorized as male-biased and female-biased (Figure 3). The 13 miRNAs that maintained female-biased expression in both seasons mainly belonged to three miRNA families: miR-10, miR-8, and miR-23. Of the let-7 family, only let-7c maintained female-biased expression in both seasons. The 50 miRNAs that maintained male-biased expression in both summer and winter primarily belonged to seven miRNA families: miR-17, miR-19, miR-34, miR-190, miR-138, miR-15, and miR-7.

### Functional Analysis of Sex-Biased miRNA Target Genes

**FIGURE 4:**
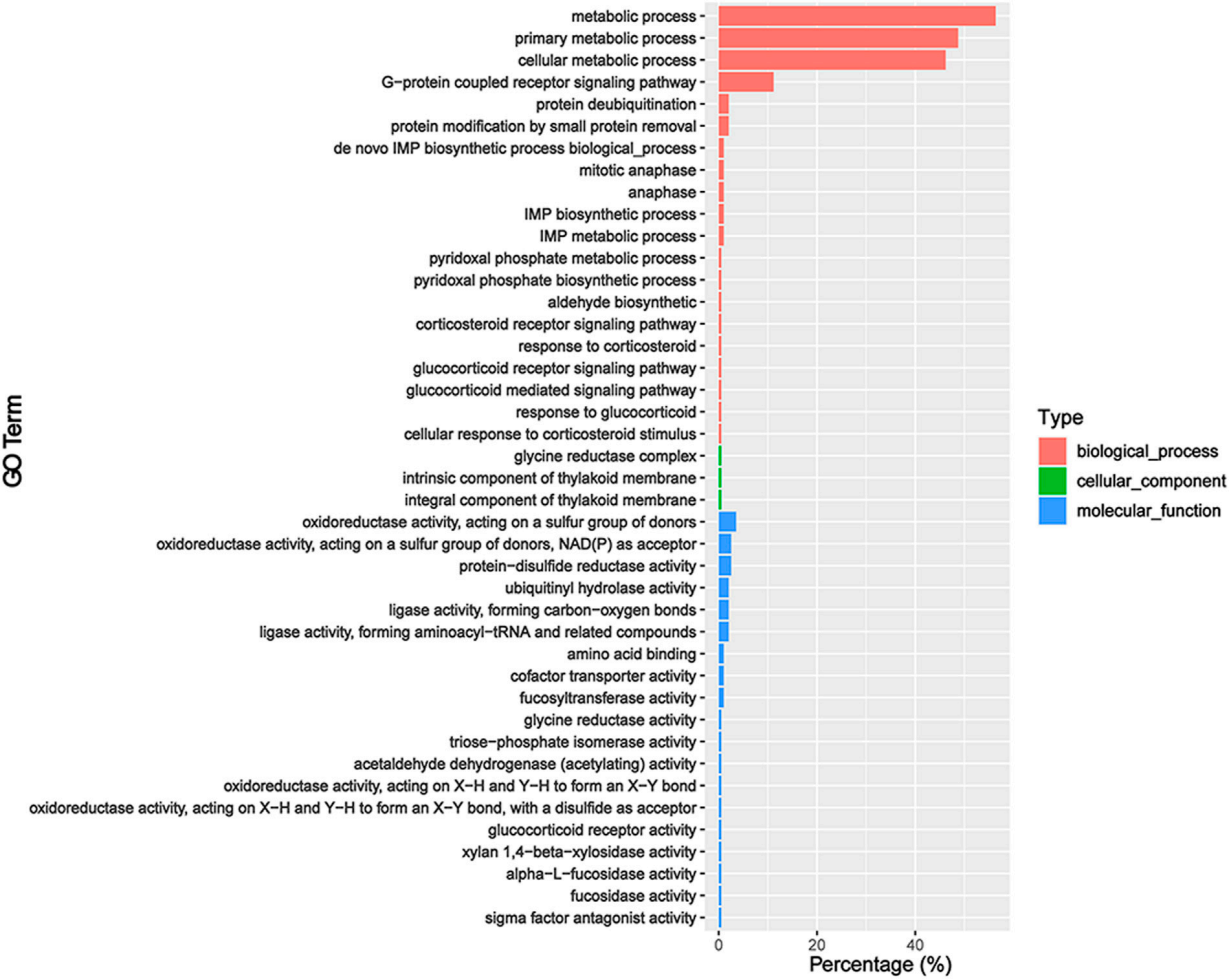
GO annotation of the predicted target genes of sex-biased differentially expressed miRNAs (ovaries relative to testes) in summer.

**FIGURE 5:**
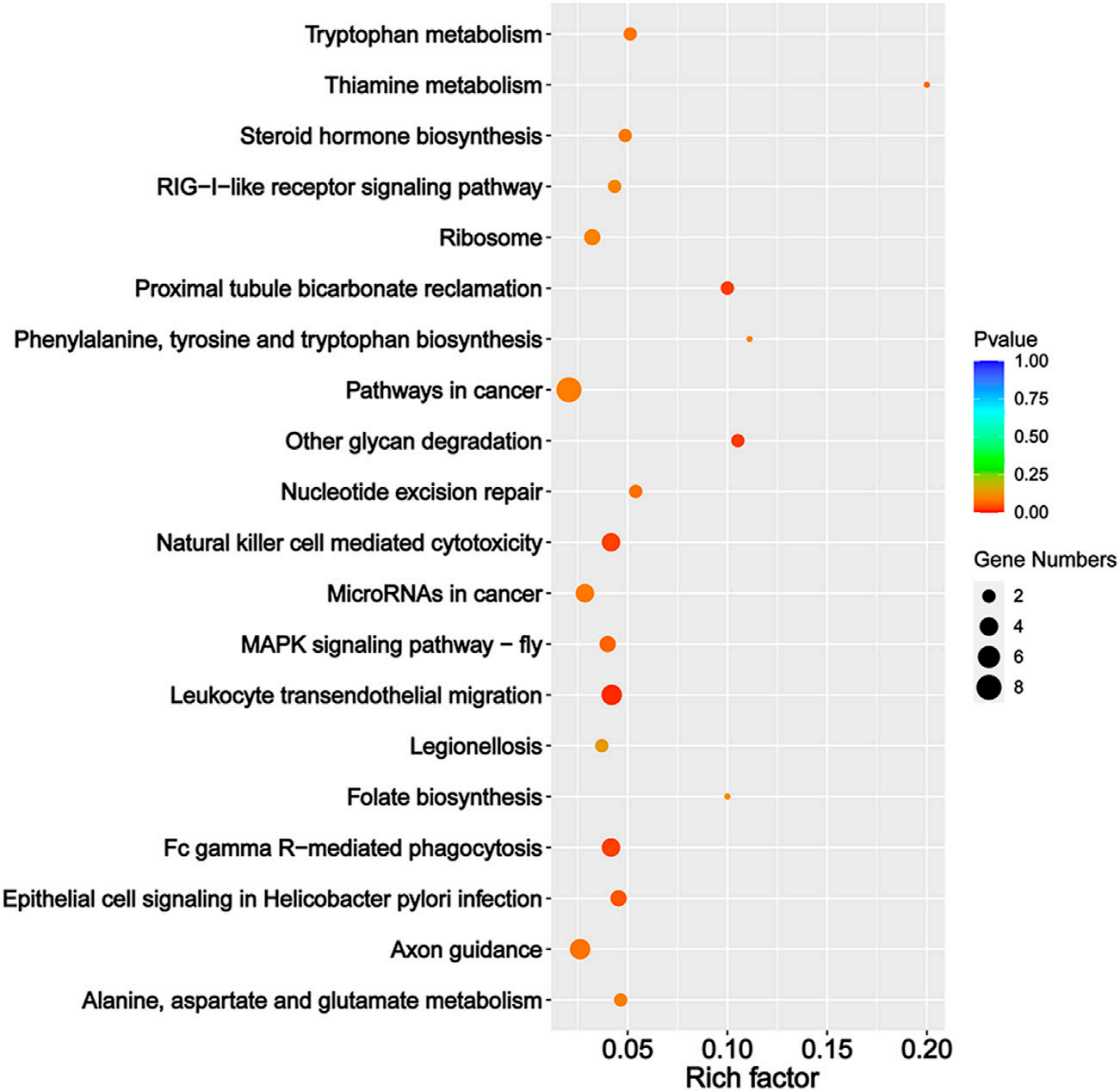
The 20 most enriched KEGG pathways of the predicted target genes of sex-biased differentially expressed miRNAs (ovaries relative to testes) in summer.

We predicted the target genes of sex-biased DEmiRs to explore their potential functions. Our analysis obtained 306 and 909 target genes for sex-biased DEmiRs in summer and winter, respectively. The target genes that were identified in summer samples were classified into 42 GO subcategories (Figure 4). Notably, six enriched GO terms (*p* < 0.05) were found to be related to steroid hormones, including corticosteroid receptor signaling pathway, response to corticosteroid, glucocorticoid receptor signaling pathway, glucocorticoid mediated signaling pathway, response to glucocorticoid, and cellular response to corticosteroid stimulus. KEGG analysis mapped these target genes to 159 pathways. One of the 20 most enriched pathways (Figure 5) was steroid hormone biosynthesis (ko00140) (*p* = 8.92 × 10^-2^). However, analysis of the winter-specific target genes revealed that no steroid-related GO terms and KEGG pathways were significantly enriched (Supplementary Figures S2, S3).

### Association Analysis of DEmiRs and DEGs

**FIGURE 6:**
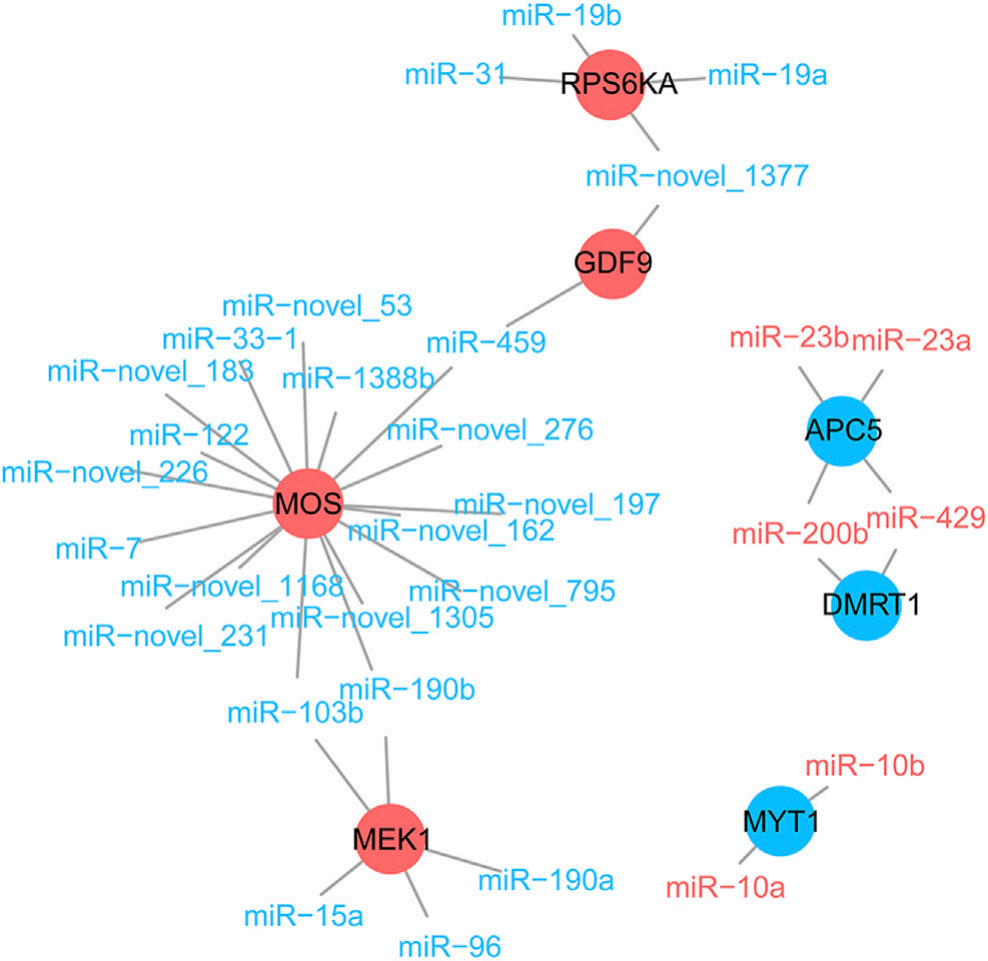
The negative regulation of some sex-related genes by differentially expressed miRNAs. Male- and female-biased miRNA are represented by blue and red fonts, while male- and female-biased differentially expressed genes are represented by blue and red circles, respectively.

**FIGURE 7:**
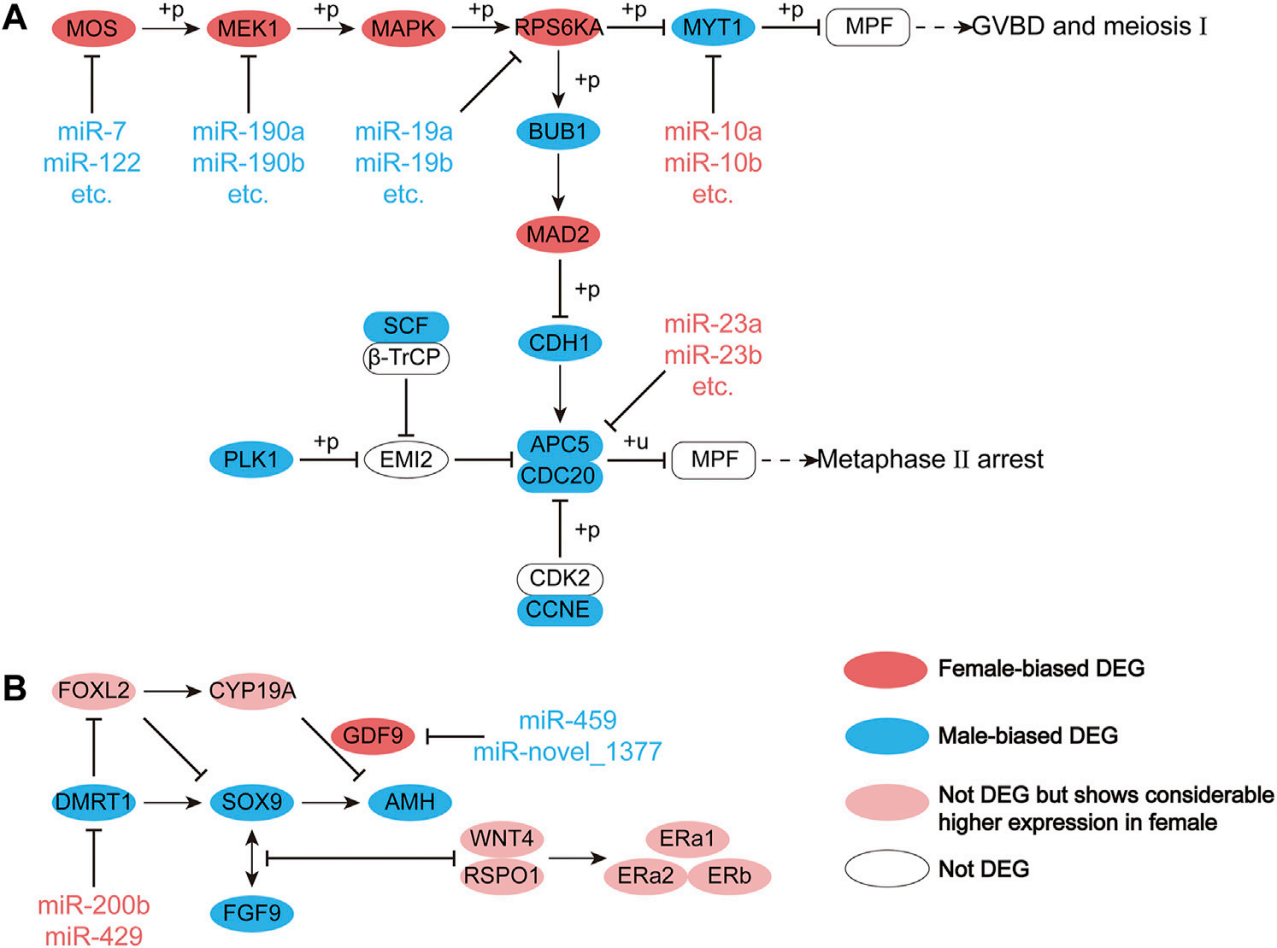
Regulation of miRNA on differential gene expression (DEG) in the **(A)** meiotic pathway of oocytes and **(B)** antagonistic gonadal function maintenance pathway during the sexual maturity period.

We explored, in more detail, the miRNAs that maintained sex-biased expression in both seasons. In silico analysis revealed that 1,659 and 2,719 genes were targeted by 13 female-biased and 50 male-biased miRNAs, respectively. Previously, we identified sex-biased differentially expressed genes (DEGs) in Chinese alligator gonads by transcriptome analysis (Lin et al., 2021). Here, we constructed interaction networks of DEmiRs and DEGs (Figure 6). Since these DEGs were associated with functional maintenance and regulation during sexual maturity, we examined these pathways in greater detail. There were five DEGs in the oocyte meiosis pathway that were targeted and regulated by DEmiRs (Figure 7A). In this pathway, three female-biased genes, *MOS*, *MEK1*, and *RPS6KA*, in the *MOS-MEK-MAPK-PSK* pathway were targeted by 17, 5, and 4 male-biased DEmiRs, respectively. While RSP6KA inhibits the expression of *MYT1* through phosphorylation, *MYT1* was negatively regulated by two female-biased miRNAs, resulting in its low expression in the ovary. Due to the suppressive effect of MYT1 on MPF, the low expression of *MYT1* activated the germinal vesicle breakdown mediated by MPF to begin meiosis I. In meiosis II, *APC5* was targeted by four female-biased miRNAs, showing significant male-biased expression, which maintained a high level of MPF expression in the ovary and achieved stagnation in the middle of the second meiosis until fertilization. There were also two DEGs in the antagonistic gonadal function maintenance pathway that were targeted and regulated by DEmiRs (Figure 7B). These two genes are an important male sex-determining gene, *DMRT1*, and a key oocyte secretion factor, *GDF9*.

## Discussion

The gonads are important reproductive organs for animals, specifically those relying on sexual reproduction, to produce gametes and steroid hormones. As an important post-transcriptional regulatory mechanism for the formation of sexual dimorphism, miRNA has been reported in the gonads of several reptiles with TSD (Lin et al., 2018; Ma et al., 2020; Xiong et al., 2020). Here, we performed sRNA sequencing to analyze gonad miRNA of adult Chinese alligators in winter and summer, and constructed miRNA expression profiles. The distribution pattern of sRNAs between the testes and ovaries was different, and this pattern was retained between seasons. In the ovarian sRNA size distribution, only a miRNA peak was observed; however, in the testes, two peaks were observed, corresponding to miRNA and piRNA. A similar pattern has been reported in the gonads of yellowfin seabream (*Acanthopagrus latus*), but the proportion of piRNA in the gonads of yellowfin seabream is larger than that of miRNA (Li et al., 2020). The high proportion of piRNA in the testes is indispensable in testicular development (Li et al., 2013; Kang et al., 2014), indicating that piRNA in the testes of Chinese alligator may play an equally critical role.

The miR-10 family is a common type of miRNA in the ovaries, and its members maintain high expression in the follicles of different species (Hossain et al., 2012; Salilew-Wondim et al., 2014; Santonocito et al., 2014). MiR-10 and miR-23 family members regulate granulosa cell apoptosis in different species (Nie et al., 2015; Jiajie et al., 2017). Since granulosa cell apoptosis is the basic physiological mechanism of follicular atresia (Zhang et al., 2019), these two family members may have a regulatory function in the follicular development cycle of female Chinese alligators. Female mice lacking miR-200b and miR-429, members of the miR-8 family, failed to ovulate normally and their fertility was greatly reduced, while males showed no change (Hasuwa et al., 2013). We observed that miR-148a maintained female-biased expression in summer and winter, and had the highest abundance in the gonads. MiR-148a is usually studied in association with cancer and is considered to be an important factor that can either promote tumorigenesis or suppress tumors (Li et al., 2016). In ovarian cancer, the high expression of miR-148a can significantly reduce the proliferation and invasion of ovarian cancer cells, and promotes cell apoptosis (Zhao et al., 2016; Zhu et al., 2019). In the ovaries of adult Chinese alligators, the high expression of miR-148a indicates the health of the ovaries. In addition, only let-7c of the let-7 family maintained its female-biased expression in both seasons, while other family members (Let-7a, 7e, and 7f) showed female-biased expression only in summer, which may be related to the hibernation habit of Chinese alligator (Lin et al., 2020).

The miR-17/92 cluster and its paralog cluster miR-106b/25 may cooperatively regulate spermatogonial cell development, and the functional loss of the miR-17/92 cluster in male mice germ cells may lead to smaller testes, reduced epididymal sperm count, and defective spermatogenesis (Tong et al., 2012). Members of the miR-34 family may also have regulatory functions in spermatogenesis or motility, and miR-34b/c and miR-449 clusters have redundant functions in the regulation of germ cell development in the mice testes (Bao et al., 2012). Adult male mice with double knockout of miR-34b/c and miR-449 clusters showed male sterility, low sperm count and motility, and deformation (Wu et al., 2014; Yuan et al., 2015). In zebrafish, another member of the miR-34 family, miR-34a, negatively regulates sperm motility by targeting *gsk3a* (Guo W. et al., 2017). In the gonads of adult Chinese alligator, miR-34b/c and miR-449a/b demonstrated male-biased expression in summer, and the expression of miR-34b and miR-34c in the testes was much higher than that of miR-449a and miR-449b. In winter, the differential expression of miR-449a/b between the gonads did not reach statistical significance, and the testicular expression level in winter was much lower than in summer. This suggests that the miR-34 family, especially miR-34b/c, has important functions in the development of male gonads and sperm in Chinese alligator, and miR-449 plays an auxiliary regulatory role in this regulatory function during the summer breeding season.

Previously, we constructed the oocyte meiosis, steroid biosynthesis, and antagonistic sex maintenance pathway in the gonads of Chinese alligator (Lin et al., 2021). However, we found that most of the target genes of sex-biased DEmiRs were concentrated in the previously constructed oocyte meiosis pathways, and a small number appear in the mutually antagonistic sex maintenance pathways, while hardly any appear in the steroid biosynthesis pathways. In the mutually antagonistic sex maintenance pathways, the genes *DMRT1* and *GDF9* were targeted by female-biased and male-biased DEmiRs, respectively. In the testes of adult mice, the lack of *DMRT1* in Sertoli cells will cause the Sertoli cells to not express *SOX9*, which promotes male gonadal development, and instead express *FOXL2*, which promotes female gonadal development, to reprogram the Sertoli cells into granular cells (Lin et al., 2021). The inhibition of *DMRT1* expression by miR-200b and miR-429 in the ovary allows the female sex to be maintained in adulthood. *GDF9* is an important oocyte secretion factor in a variety of vertebrates, and plays a role in the stability of the oocyte microenvironment and the maintenance of the quality of the oocyte (Gilchrist et al., 2008; Chen et al., 2017; Liu et al., 2018). In an *in vitro* experiment to study zebrafish gonadal differentiation, *GDF9* could significantly inhibit *AMH* and co-express with *CYP19A* in gonadal differentiation (Chen et al., 2017). *CYP19A* is a downstream regulatory gene of *FOXL2*, and can encode aromatase to irreversibly convert testosterone into estradiol and promote the functional maintenance of the ovary (Wang et al., 2007; Lance, 2009). Inhibition of *GDF9* by miR-429 and miR-novel_1377 in testes maintained male sex in adulthood. The targeted regulation of miRNAs on *DMRT1* and *GDF9*, therefore, enables the maintenance of male and female sex.

In order to explore the difference between adult and embryonic gonads of the Chinese alligator, we compared the expression of miRNA in adult and embryonic gonads (Lin et al., 2018) and observed that the target genes differed between the developmental stages. Two miRNAs of the miR-10 family (miR-10a/b) showed female-biased expression in mid-TSP and adulthood, but the target genes were different. In mid-TSP, miR-10a and miR-10b targeted two male sex-differentiation genes, *ADCY4* and *FGFR2*, while in adulthood, the targeted gene was *MYT1*, which has important functions in the oocyte maturation. Two members of the miR-133 family (miR-133a/b) had sex-biased expression in both post-TSP and adulthood, but the biased expressions were not consistent. MiR-133b had male-biased expression in both post-TSP and adulthood in winter; miR-133a showed male-biased expression in post-TSP, but in adulthood, it had opposite sex-biased expression in winter and summer. In addition, in post-TSP, these two miRNAs targeted multiple genes related to sex determination and sex differentiation, such as *StAR*, *CYP11A*, *HSD17B6* and *MAP3K1*, while in adult stage, the only sex-related gene targeted by these two miRNAs is *StAR*. In Chinese alligators, miR-133a and miR-133b have important sex-determination functions in the embryonic stage, but in adulthood their sex-related functions were reduced. This suggests that the same miRNA may regulate different genes and play different roles at different stages of animal development.

## Conclusion

We identified miRNAs in the gonads of Chinese alligators, analyzed the differential expression patterns between testes and ovaries, and further investigated the role of miRNAs in Chinese alligator sex determination, differentiation, and maintenance. These results will further inform our understanding of the epigenetic mechanisms of the formation and maintenance of sexual dimorphism. We anticipate that this research will contribute to the conservation of the Chinese alligator, a particularly endangered species.

## Data Availability

Publicly available datasets were analyzed in this study. The Chinese alligator reference genome is available from GenBank (assembly accession: GCA_000455745.1). The sRNA-seq data used in this work has been deposited in the NCBI SRA database under BioProject accession numbers PRJNA556092.
