# Supplementary material for: Sex-Biased miRNAs in the Gonads of Adult Chinese Alligator (Alligator sinensis) and Their Potential Roles in Sex Maintenance
Source: Front Genet. 2022 Mar 31;13:843884. doi: 10.3389/fgene.2022.843884 (PMC9008718; doi:10.3389/fgene.2022.843884)
Supplement: Supplementary file 1 [file DataSheet1.docx]

***Supplementary Material***

# Supplementary Tables

**Supplementary Table S1** Summary of the sRNA sequencing date.

| **Sample** | **S_OVA** | **S_TES** | **W_OVA** | **W_TES** | **Total** |
| --- | --- | --- | --- | --- | --- |
| **Raw Reads** | 13,292,653 | 13,604,474 | 11,777,565 | 13,029,475 | 51,704,167 |
| **Clean Reads** | 13,075,837 | 13,313,795 | 11,541,190 | 12,766,605 | 50,697,427 |
| **Read 18-35 nt** | 12,654,157 | 12,371,847 | 11,251,236 | 10,894,300 | 47,171,540 |
| **Uniq Reads** | 244,343 | 2,328,328 | 229,832 | 2,657,358 |  |
| **Mapped Reads** | 11,787,824 | 11,052,163 | 10,635,853 | 9,737,335 | 43,213,175 |

**Supplementary Table S2** Category of sRNA in gonads of adult Chinese alligator.

| **Sample** | **S_OVA** | **S_TES** | **W_OVA** | **W_TES** | **Total** |
| --- | --- | --- | --- | --- | --- |
| **Mapped Reads** | 11,787,824 | 11,052,163 | 10,635,853 | 9,737,335 | 43,213,175 |
| **rRNA** | 81,897 | 153,893 | 51,583 | 205,943 | 493,316 |
| **tRNA** | 2 | 10 | 8 | 20 | 40 |
| **snRNA** | 1,810 | 11,892 | 2,625 | 38,182 | 54,509 |
| **snoRNA** | 963 | 2,525 | 1,764 | 2,849 | 8,101 |
| **Repeat** | 94,893 | 1,340,965 | 103,763 | 1,149,621 | 2,689,242 |
| **Novel miRNA** | 7,050,753 | 3,688,877 | 7,191,673 | 2,173,034 | 20,104,337 |
| **Exon** | 94,646 | 625,866 | 135,804 | 716,701 | 1,573,017 |
| **Intron** | 124,749 | 850,765 | 147,634 | 814,415 | 1,937,563 |
| **Other** | 4,338,111 | 4,377,370 | 3,000,999 | 4,636,570 | 16,353,050 |

**Supplementary Table S3** Summary of miRNA prediction.

| **Sample** | **S_OVA** | **S_TES** | **W_OVA** | **W_TES** |
| --- | --- | --- | --- | --- |
| **Mature miRNA** | 558 | 674 | 424 | 547 |
| **Star miRNA** | 212 | 250 | 159 | 192 |
| **miRNA hairpin** | 581 | 705 | 438 | 571 |
| **Uniq miRNA** | 2,718 | 3,520 | 2,105 | 2,891 |
| **Total miRNA** | 7,050,753 | 3,688,877 | 7,191,673 | 2,173,034 |

# Supplementary Figures


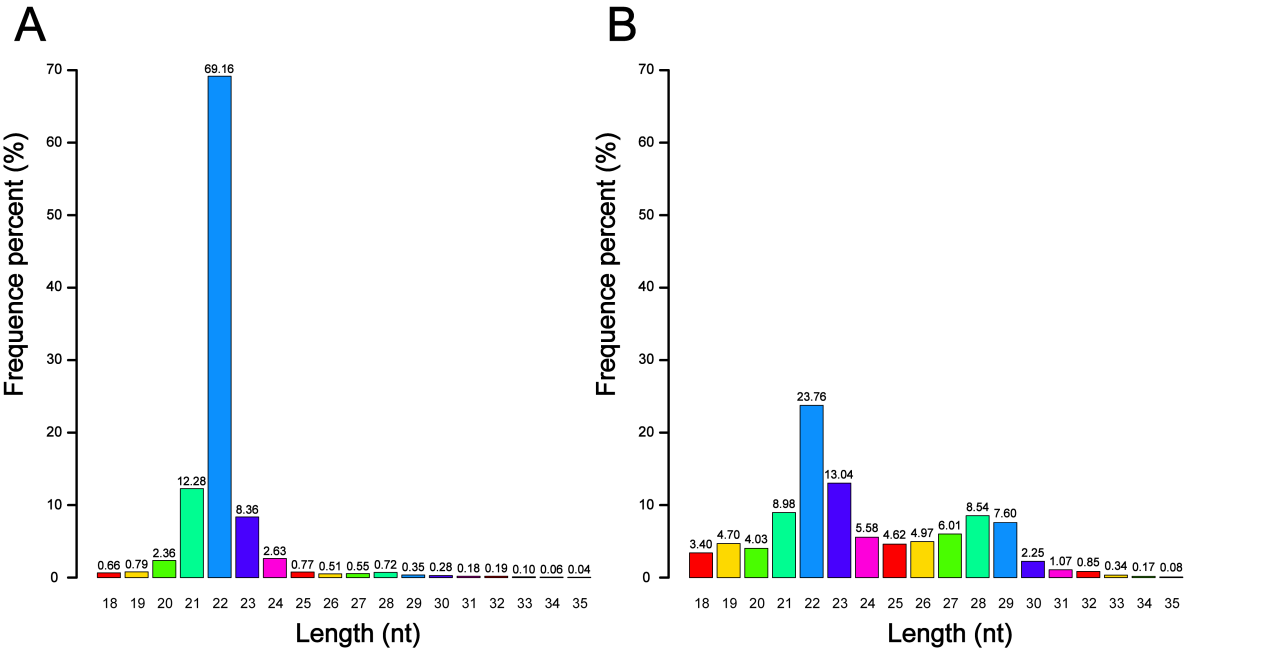


**Supplementary Figure S1.** The nucleotide (nt) length distribution of small RNA in adult Chinese alligator ovaries (A) and testes (B) sampled in winter.


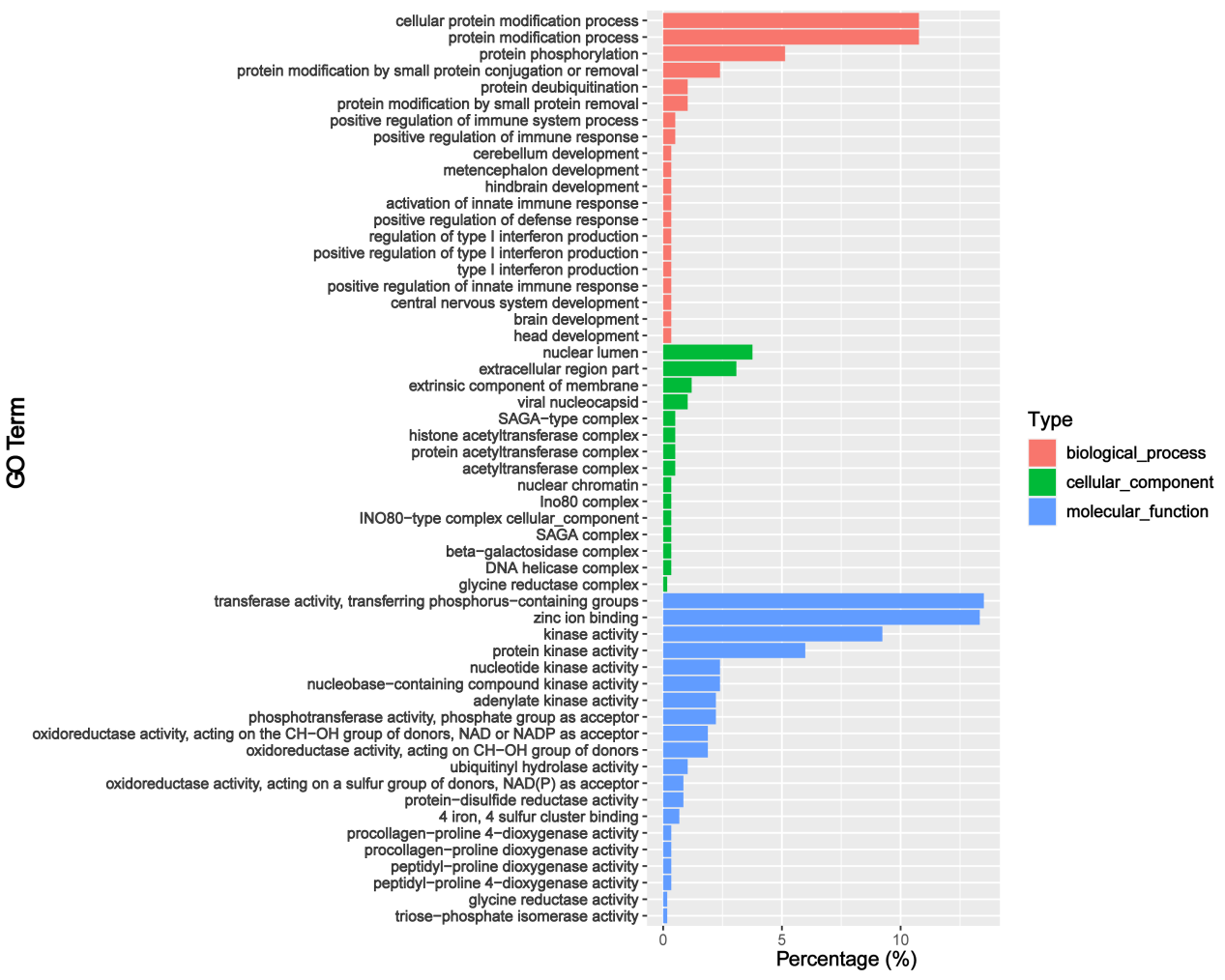


**Supplementary Figure S2.** GO annotation of the predicted target genes of sex-biased differentially expressed miRNAs (ovaries relative to testes) in winter.


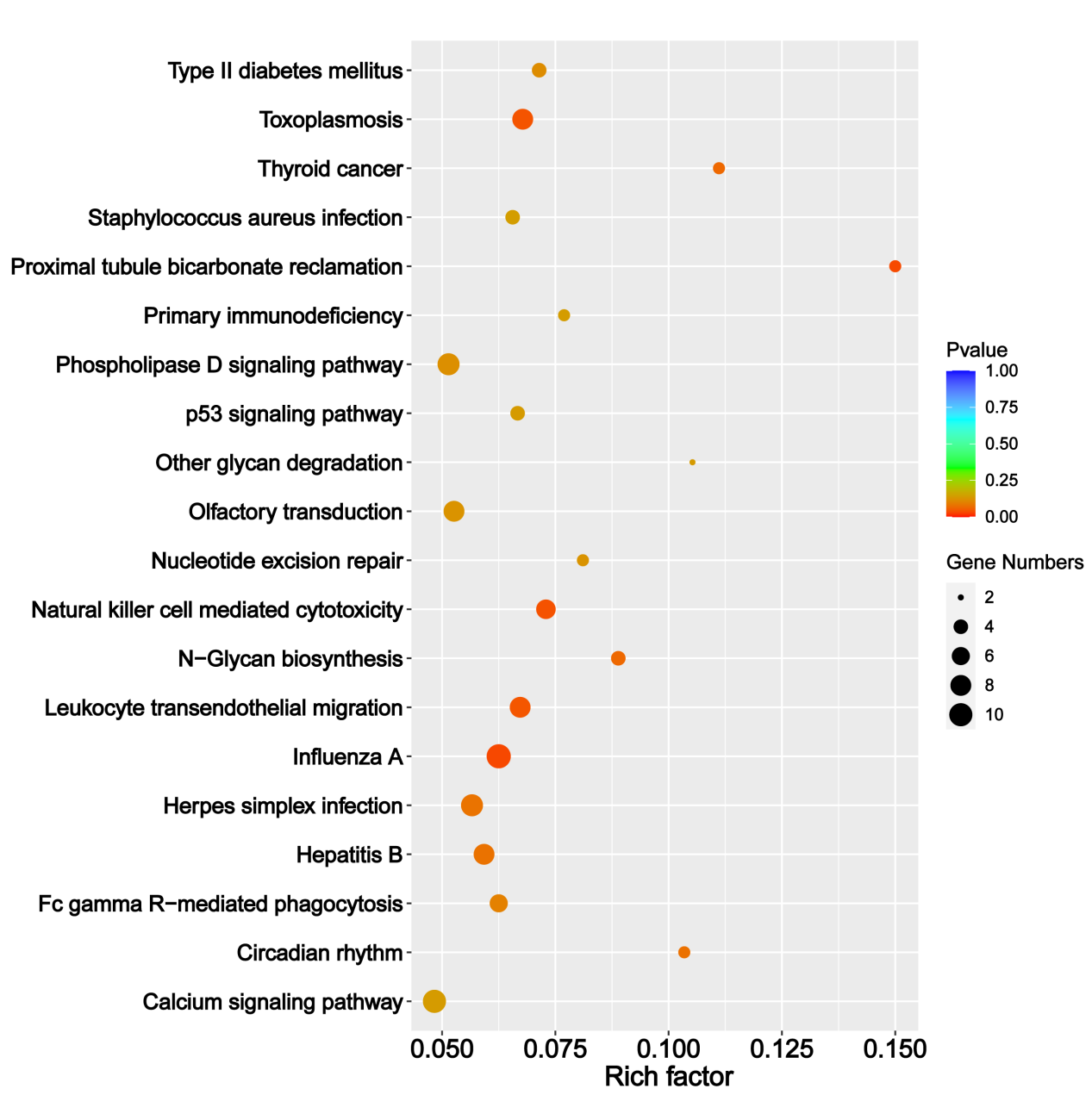


**Supplementary Figure S3.** The 20 most enriched KEGG pathways of the predicted target genes of sex-biased differentially expressed miRNAs (ovaries relative to testes) in winter.
